# Supplementary material for: Cost-utility analysis of Cryoballoon ablation versus Radiofrequency ablation in the treatment of paroxysmal atrial fibrillation in Iran
Source: PLoS One. 2022 Jul 6;17(7):e0270642. doi: 10.1371/journal.pone.0270642 (PMC9258804; doi:10.1371/journal.pone.0270642)
Supplement: S1 Table — (DOCX) [file pone.0270642.s001.docx]

**Table S1: Probability of death at different ages (Iran Life Table)**

| Cycle (year) | Age | P_Death |
| --- | --- | --- |
| 1 | 50 | 0.0037 |
| 2 | 51 | 0.00402 |
| 3 | 52 | 0.00434 |
| 4 | 53 | 0.00466 |
| 5 | 54 | 0.00498 |
| 6 | 55 | 0.0053 |
| 7 | 56 | 0.00614 |
| 8 | 57 | 0.00698 |
| 9 | 58 | 0.00782 |
| 10 | 59 | 0.00866 |
| 11 | 60 | 0.0095 |
| 12 | 61 | 0.01116 |
| 13 | 62 | 0.01282 |
| 14 | 63 | 0.01448 |
| 15 | 64 | 0.01614 |
| 16 | 65 | 0.0178 |
| 17 | 66 | 0.0208 |
| 18 | 67 | 0.0238 |
| 19 | 68 | 0.0268 |
| 20 | 69 | 0.0298 |
| 21 | 70 | 0.0328 |
| 22 | 71 | 0.0388 |
| 23 | 72 | 0.0448 |
| 24 | 73 | 0.0508 |
| 25 | 74 | 0.0568 |
| 26 | 75 | 0.0628 |
| 27 | 76 | 0.06822 |
| 28 | 77 | 0.07364 |
| 29 | 78 | 0.07906 |
| 30 | 79 | 0.08448 |
