# Supplementary material for: Cost-utility analysis of Cryoballoon ablation versus Radiofrequency ablation in the treatment of paroxysmal atrial fibrillation in Iran
Source: PLoS One. 2022 Jul 6;17(7):e0270642. doi: 10.1371/journal.pone.0270642 (PMC9258804; doi:10.1371/journal.pone.0270642)
Supplement: Attachment — Submitted filename: response to reviewers.docx [file pone.0270642.s002.docx]

**Reviewer 1 Comments:**

**Dear reviewer**

Thank you for your invaluable comments and guidance to improve our manuscript.

We presented the responses one by one on your comments as follows:

Reviewer #1: General comments:
This paper focusses on the cost-effectiveness of two alternative treatment methods (Cryoballoon Ablation versus Radiofrequency Ablation) for paroxysmal Atrial Fibrillation. I would like to thank the authors for their work. I think the study is interesting and it can be brought to a publishable level after some revisions.
I think the authors need to use a checklist to ensure a high-quality standard of reporting, such as CHEERS. More details and justification needed when explaining the model and the parameters. I think the probabilistic sensitivity analyses need to be repeated as I have some concerns over the choice of comparator. There are also some inconsistencies between the Figures and the text. I think some the tables need to be re-organised as well. I explain these below.
The text needs to be updated to improve the flow and it should be grammatically checked. The authors may find using online applications (such as Grammarly) helpful. There is repetition at some places, so please make sure there is not two different sentences with the same meaning. Please also add page numbers to the manuscript.

Response: Thanks for your invaluable comments. Final manuscript was revised by native English editor. We matched the study reports and findings with the CHEERS checklist. Also, we applied other specific comments as much as possible.

Specific comments:
Page 9 – Abstract:
Line 51. Please provide a reference here.

Response: It was revised based on your comment.

Line 52. "should be of which"

Response: Thanks for your comment. It was revised based on your comment.

Line 53. Please add paroxysmal before AF to be clear. I also suggest removing "analysis" and inserting "the" before cost utility.

Response: Thanks for your comment. It was revised based on your comment.

Line 60. Please remove the word “rather” as it is not used to make comparisons.

Response: It was removed.

Line 61. I think "one time" unnecessary here since when it is just the GDP we understand that it is one time of the GDP.

Response: Thanks for your comment. It was removed based on your comment.

Line 62. Please remove “but” to avoid redundancy

Response: It was revised based on your comment.

Lines 63 and 65. It sounds as if PSA and Monte Carlo simulation are two different analyses while in fact, they refer to the same analysis. I suggest re-writing this sentence. Also, instead of saying "by probability of 60%" it might be clearer to say, "the probability of cost-effectiveness was 60%."

Response: Thanks for your comment. It was revised based on your comment.

Page 10 – Introduction:
I think in the introduction, there is too much focus on the clinical aspects and too little on the cost-effectiveness. The authors need to explain what we already know about the cost-effectiveness of CBA and RFA then tell us about the limitations of existing economic models and why a new model was needed.

Response: Thanks for your comment. It was revised based on your comment. We explain results of some similar studies in “Introduction” section. In Iran, as mentioned in the text, no full economic evaluation study has been conducted to compare these two technologies, and present study is the first study in Iran in this regard. Due to the fact that the results of similar economic evaluation studies are different in different contexts, the present study was conducted for the first time in Iran.

Line 73. It is hard to follow this sentence so, I suggest dividing into two or three sentences.

Response: It was revised based on your comment.

Page 11
Line 115. Please, here and everywhere in the text, add "paroxysmal" to be clear

Response: Thanks for your comment. It was revised based on your comment.

Page 12
Line 121. This guideline is not for health projects but for health technologies.

Response: Thanks for your comment. It was revised.

Line 124. I think the authors refer to the design of the economic model - I'd update this sentence as “The economic model was designed based on a literature review ...”

Response: Thanks. It was revised based on your comment.

Line 130. The word “observed” is not meaningful here it should be replaced with a word like “demonstrated”.

Response: Thanks for your comment. It was revised based on your comment.

Line 132. For consistency, please consider updating the first state's name to "before intervention" or the word "intervention" in the other states to "ablation".

Response: Thanks for your comment. It was revised based on your comment.

Lines 136 and 138. Please re-phrase this sentence to be clear and I'd suggest starting it with "It was assumed that..."

Response: Thanks for your comment. Sentences revised based on your comment.

Page 13
Figure 1. I think the model demonstration in Figure 1 doesn't match the description in the text.

Response: Description Sentences was revised. Figure 1 and related description was matched.

I think the authors should draw another tree. For example, one of the health states is Atrial Fibrillation I think this should be pre-intervention. Similarly, it is confusing to see CBA just after Atrial Fibrillation but not RFA.

Response: Thanks for your comment. Given that the structure of the model is similar in the two comparison arms, in Figure 1 we only included the model structure of one arm. Also, we revised the health state name from “Atrial Fibrillation” to Pre-intervention Atrial fibrillation. Please See revised Fig 1.

In the methods section, the authors need to explain and justify why specific complications were chosen and not the others.
Additionally, the price year needs to be stated in the methods section.

Response: It was revised based on your comment. As mentioned in the text (method section) we included “pericardial effusion” , “cardiac tamponade”, “permanent phrenic nerve palsy”, “vascular complications” and “Stroke” as side effects of the technologies in the model, which we considered both their related cost and the disutility values. The choice of side effect in present study was based on the evidence from previous studies and also in consultation with the clinical specialists.

Line 139. Evidence is a singular word: we cannot write evidence. Please correct this everywhere

Response: It was revised based on your comment.

Line 140. This needs to be explained more which hospital? The hospital that you took the cost data. You may also cite the existing studies here. Additionally, please rephrase this sentence.

Response: Thanks for your comment. It was revised based on your comment and highlighted.

Lines 152 and 153. I don't understand this sentence: could you please rephrase?

Response: It was revised.

Line 163. This needs to be supported with published evidence.

Response: Thanks for your comment. It was revised based on your comment.

Line 168. Is it publicly available? If so, please provide a reference or provide a summary table as an Appendix.

Response: Thanks for your comment. All the parameters, values and evidence used are given in Table 1.

Table 1. The authors state following the NICE recommendations; however, they used a discount rate of 0.05. This should be explained and justified.

Response: We used the NICE reference case to conduct economic evaluations. Only regarding the discount rate, considering the context of Iran, we decided to use a higher discount rate. However, we considered lower and higher values in the sensitivity analysis.

Table 1 includes mortality during first year, but it is not clear what probability was used after the first year. The authors need to explain this in the text.

Response: Thanks for your comment. As you know, the probability of mortality increases with age and this issue is considered in Markov model using table. In Table 1, we only mentioned the probability of death in the first year. We can include the entire probability of death table (Iran Life Table) as S1 in Supporting Information. Please see S1 in Supporting Information.

Table 1 reports the probabilities for complications under the title of “transition probabilities” and this is really confusing. I think Table 1 needs to be re-organised such that the transition probabilities and morbidity risks are clear.

Response: Table 1 revised based on your comment.

The SD or CI should be provided for all the probabilities

Response: Thanks for your comment. We provide SD or CI for variables which were used in sensitivity analysis only. SD or CI for other variables were not used for other variables in the model.

What is a life table? Please cite a reference or provide this table as an Appendix.

Response: Thanks for your comment. As mentioned above, we include the entire probability of death of normal population (Iran Life Table) as S1 in Supporting Information. Please see S1 in Supporting Information. Also, reference was added in table 1.

In Table 1 some parameters were identified through “survey and calibration” this needs to be explained in the text. What do you mean when you say survey and calibration? We need to see the original figures/values and those used in the model after adjustments.
Response: Thanks for your comment. By “survey and calibration” we mean that the values are not used from another reference and are obtained in the present study. But to avoid misunderstandings we replaced it with “our study” in table 1.

Page 16
Line 189. Citation needed here. I think WHO recommends using three times of GDP as the threshold. Please explain why you have chosen to use the GDP. Please see this paper on the thresholds recommended by WHO <https://www.who.int/bulletin/volumes/93/2/14-138206.pdf>

Response: Thanks. We added reference. WHO recommended 1 to 3 times the per capita GDP for cost effectiveness threshold in developing countries.

Also, the following articles have reviewed the threshold recommended by who, saying that three times the per capita GDP as a cost-effectiveness threshold is high. Please see:

1. Bertram MY, Lauer JA, De Joncheere K, et al. Cost-effectiveness thresholds: pros and cons. Bull World Health Organ. 2016;94(12):925–930.
2. Marseille E, Larson B, Kazi DS, Kahn JG, Rosen S. Thresholds for the cost–effectiveness of interventions: alternative approaches. Bull World Health Organ. 2015;93(2):118–124.

Line 200. This needs to be more specific. 10%, 20% or a range of values between 10% and 20%?

Response: As mentioned in manuscript about PSA, in cases which no evidences regarding variance of the variable were found, 10-20% of mean values of variable was considered as standard deviation. In some cases 10% and in some cases 20%.

Line 201. Please provide a reference here to show how you have chosen the appropriate distributions.

Response: Thanks for your comment. As mentioned in manuscript, the range used for uncertainty, statistical distributions and related reference of each variable used in PSA are presented in table 1. In cases which no evidences regarding variance of the variable were found, 10-20% of mean values of variable was considered as standard deviation. In some cases 10% and in some cases 20%.

Page 17
Line 204. Please use commas for costs to make sure these are clear. For example, is it $14,198.3?

Response: Thanks for your comment. It was revised based on your comment.

Table 2. Please re-organise this table and report the incremental figures in a separate raw. Also, please change the column name from Eff** to QALYs to be more specific.

Response: It was revised based on your comment. Please see revised Table 2.
Page 18
Figure 3. This figure is confusing because the impact on the net benefit was demonstrated which was not mentioned anywhere in the text. Please repeat this analysis to show the impact of changing inputs on ICER calculations. Additionally, we cannot tell which lower and upper values were used. So, please provide these on the graph. Please re-label the parameters and try not to use abbreviations. Please
also explain what EV represents. Again, use commas for costs.

Response: Thanks for your comment. It was revised based on your comment. Also, we mentioned EV (Expected Value) represents ICER. Please see revised figure 3. Also, commas were used in whole text for costs.

Figures 4 and 5. Please repeat these analyses using ICERs not net benefits.

Response: Thanks for your comment. We repeat the sensitivity analysis again based on your comment. Please see revised figure 3.

Line 242. Why 1000 times? why not 5000 or 10000? This needs to be justified.

Response: Thanks for your comment. As you know, there is no specific reference to how many times we do the sampling in monte-carlo simulation, and researchers usually determine this amount based on the study, which is usually considered 100 or 1000 times in most studies. But in order to be more precise in this regard, we repeat the monte-carlo simulation by 10,000 times sampling. Please see.

Line 243. This sentence raises concern. It was said that the cost-effectiveness of CBA would be estimated compared to RFA. However, the PSA was conducted to estimate the cost-effectiveness of CBA and RFA compared to something else which was not explained to the reader. Is the probability of CBA being cost-effective compared to RFA 59% as stated in the abstract or is the probability of CBA being cost-effective compared to something else is 59%? The authors need to clarify this and repeat the analysis to compare CBA to RFA.

Response: We definitely meant that the probability of CBA being cost-effective compared to RFA is 59% based on strategy selection diagram. Of course, we deleted this chart based on reviewers’ comment at this stage. We reported the PSA findings solely on the basis of the Scatter plot and 10,000 iterations.

Page 19
Figure 7. This figure is in line with the text, so maybe use this on and remove Figure 6 and related text. Here, please change effectiveness to QALYs.

Response: It was revised based on your comment.

Table 3. I don't think this makes sense. We don't need this table, just remove it please.

Response: Thanks for your comment. We reported this table to determine the exact percentage placement of interventions in each quadrant of the cost-effectiveness plane. However, if you insist on this comment, we can delete this section in the next step.

Page 20
Lines 262 and 265. This part should be moved to introduction.

Response: Thanks. We would like this section not to be removed from the discussion. In fact, we used this as an introduction to this section to compare the effectiveness and cost-effectiveness of technologies. However, if you insist on this comment, we can delete this section in the next step.

Line 269. Please don't repeat the results here - it would be enough just to say that it was not cost-effective at a WTP of $7,142 but cost-effective at a WTP of $14,285.

Response: Sentences was revised based on your comment.

Line 279. Why would the cost of RFA increase? Please rephrase this as "the cost of RFA is $5,738 not $5,127 ..."

Response: We rephrased it. Please see.

Line 282. I don't understand this phrase (re-use of necessities). Please explain what this means.

Response: We revised this phrase. Please see discussion.

Page 21
Line 298. We wouldn't say viewpoint to describe the perspective in economic evaluations.

Response: Thanks for your comment. It was revised based on your comment.

**Reviewer #2:**

Thank you for your invaluable comments and guidance to improve our manuscript.

We presented the responses one by one on your comments as follows:

Please remove "Case Study" from the title.

Response: Thanks for your comment. It was revised based on your comment.

English language of the manuscript needs to be improved.

Response: Thanks for your comment. Manuscript was revised by native English editor.

Proof-editing the manuscript because grammar, punctuation and use of acronyms is rather poor.

Response: Thanks for your comment. Manuscript was revised by native English editor.

Please check keywords with Mesh terms.

Response: It was revised based on your comment.

The authors should harmonize the cost-utility/ cost utility in the manuscript.

Response: Thanks for your comment. Manuscript was revised based on your comment.

Lines 188-189: please add some references.

Response: Thanks for your comment. Reference was added.

Line 190: TreeAge 2011 software/ line 59: TreeAge pro 2020 software. Please correct.

Response: Thanks for your comment. It was revised. We used TreeAge pro 2020 software.
