# Supplementary material for: Cost-utility analysis of Cryoballoon ablation versus Radiofrequency ablation in the treatment of paroxysmal atrial fibrillation in Iran
Source: PLoS One. 2022 Jul 6;17(7):e0270642. doi: 10.1371/journal.pone.0270642 (PMC9258804; doi:10.1371/journal.pone.0270642)
Supplement: Attachment — Submitted filename: response to reviewers r2.docx [file pone.0270642.s003.docx]

**Response to reviewers: Round 2**

**Reviewer 1 Comments:**

**Dear reviewer**

Thank you for your invaluable comments and guidance to improve our manuscript.

We presented the responses one by one on your comments as follows:

Reviewer #1: General comments:
I thank the authors for their work, and I think the manuscript has been improved significantly. I have some additional comments.

A final, through proof-reading is needed since there are some grammatical errors (for example tense inconsistencies) and several sentences that could be improved. Add page numbers, please.

Response: Thanks for your comment. Another proof-reading was conducted.

Introduction

Line 74 – Please replace the word “pass” with “follow”.

Response: It was revised based on your comment.

Line 106 – Please replace the word “various” with “some”.

Response: It was revised based on your comment.

Line 116 – Please rephrase this sentence as follows: “Because CBA is a relatively new technology in Iran, …”

Response: Thanks for your comment. It was revised based on your comment.

Figure 1 – Please make a note at the bottom of the Figure to state that the structure is the same for the control arm.

Response: Thanks. It was revised based on your comment.

Line 152 – Please write QALY in full here since it is the first time that it is been used. So, it should be: “quality adjusted life years (QALYs)”

Response: Thanks. It was revised based on your comment.

Please write the discount rate here and explain why you used 5% instead of 3% as suggested by NICE. Is there a guideline in Iran which suggests that 5% should be used?

Response: We did not have a guideline for this purpose in Iran until the study. We used 5% as discount rate for both costs and QALYs in the model based on the recommendation of the Health Technology Assessment Office of the Iran’s Ministry of Health for this study. This descriptions was also added in the Methods section of the manuscript.

The price year must be provided in the methods section. Are these 2021 $?

Response: Thanks for your comment. We mentioned in line 175 of the manuscript that “All costs were calculated based on 2019-20 prices”. Please see.

Please provide the “uncalibrated” figures and the “calibrated” figures. This way the reader can tell if the authors’ calibrations were correct. I’d call them adjustments rather than calibrations.
Response: Thanks for your comment. As mentioned in the previous correspondence, by “survey and calibration” we mean that the values are not used from another reference and are obtained in the present study. But to avoid misunderstandings we replaced it with “our study” in table 1.

Also, in cases where calibration is included with a reference as a variable source, the reader can see the original value of the variable by referring to the reference. Also, we replaced calibration with adjustment.

Line 197 – I think this sentence is abundant given that the next sentence clearly explains which WTP was used.

Response: It was revised based on your comment.

Line 211 – Please replace “10-20%” with “10% or 20%” for clarity.

Response: Thanks. It was revised based on your comment.

Figure 3 – Please refrain from using EV instead of ICER because it might be confused with another type of analysis (expected value of information). Please only use ICER.

Response: It was revised based on your comment.

Discussion

Most of the clinical and the cost data came from a study conducted in China. I think this is an important limitation and should be acknowledged. Please discuss the potential impacts of this. How can decision-makers be sure that these results are applicable to Iran? Please also discuss the potential impact on health inequalities.

Response: Thanks for your comment. As you know, the use of clinical evidence from past studies is very common in economic evaluation studies and is not usually considered as a limitation. In present study we used the best present evidence of international studies for clinical parameters, and for cost items we used internal evidence. As mentioned in the text, although in many cases there is no significant difference between different contexts regarding clinical evidence, but this is one of the possible limitations of this study that can be effective in final results. However, to eliminate this possible limitation as much as possible, in present study we performed sensitivity analysis to a wide range for uncertain parameters.
